# Can good neighbourhood perception magnify the positive effect of favourable built environment on recreational walking in China?

**DOI:** 10.1186/s12889-024-19539-x

**Published:** 2024-07-29

**Authors:** Huagui Guo, Yayu Li, Yufei Liu, Shuyu Zhang, Yanji Zhang, Hung Chak Ho

**Affiliations:** 1 School of Architecture and Urban-rural Planning, Fuzhou University, Fuzhou, 350108 China; 2 Laboratory of Smart Habitat for Humanity, Fuzhou University, Fuzhou, 350108 China; 3 School of Architecture and Urban-rural Planning, Fujian University of Technology, Fuzhou, 350118 China; 4 School of Humanities and Social Sciences, Fuzhou University, Fuzhou, 350108 China; 5 Department of Public and International Affairs, City University of Hong Kong, Hong Kong, 999077 China; 6 Social Determinants of Health Initiative, City University of Hong Kong, Hong Kong, 999077 China

**Keywords:** Perception of safety, Perception of aesthetics, Built environment, Walking for recreation, Moderation effect

## Abstract

**Background:**

It remains unknown whether good neighbourhood perception can enhance the benefits of favourable built environment to physical activity. Moreover, the moderation pattern is less understood in developing countries.

**Objectives:**

This work aims to examine the moderation effects of perceived neighbourhood safety and aesthetics on the relationship between built environment and time for recreational walking.

**Methods:**

We performed the examination using a sample of 760 residents in Fuzhou City, China. The Negative Binomial Regression Model was developed to examine the moderation roles of neighbourhood safety and aesthetics on the impact of built environment, adjusting for the effects of location, socioeconomic, personal preferences and social environment factors. Moreover, two sensitivity analyses were performed to test whether the moderators found are robust to the control of residential self-selection, and differential measures of conceptually-comparable aspects of built environment.

**Results:**

We found stronger associations of time for recreational walking with road density and proportion of parks and squares POIs for residents with high perception of neighbourhood safety, compared to those with low perception of neighbourhood safety. There was a greater effect of the proportion of parks and squares POIs, when perceived aesthetics was high than when perceived aesthetics was low. The findings of neighbourhood safety and aesthetics as moderator, were robust in the two sensitivity analyses. No significant moderation effect was found for land use diversity.

**Conclusions:**

High perceived neighbourhood safety can magnify the positive effects of road connectivity and accessibility to parks and squares. Neighbourhood aesthetics positively moderates the association of time for recreational walking with accessibility to parks and squares. The findings emphasize the need to consider safety- and aesthetics-specific differences in estimates of built environment effects. Improvements in neighbourhood safety and aesthetics are key to effective interventions in built environment to better promote physical activity.

**Supplementary Information:**

The online version contains supplementary material available at 10.1186/s12889-024-19539-x.

## Introduction

Physical activity confers great benefits to the physical and mental health of human beings. A number of studies have demonstrated the positive effects of favourable built environment on physical activity [1–3]. However, there are great variations in effect estimates across existing studies [4–6]. One potential explanation for the differential effects, may come from environmental moderators such as neighbourhood safety from crime and aesthetics, which can moderate the effect of built environment. An in-depth understanding of the moderating effects of these neighbourhood characteristics, is not only an important methodological issue for built environment-human health research [7], but also informative to the making of effective interventions in built environment to well promote human healthy behaviours and actions. Hence, it is of great significance to examine the moderation effect of neighbourhood safety and aesthetics.

Several studies have investigated the complex associations between built environment, neighbourhood safety and aesthetics, and physical activity. The 5Ds framework is widely used to measure built environment, including density, diversity, design, destination accessibility, and distance to transit [8]. Studies have indicated that the availability and accessibility to public facilities, such as parks usually operationalized as distance to the nearest park, are positively associated with recreational walking and moderate-to-vigorous physical activity (MVPA) [1, 9]. Road intersection density are more likely to promote walking behaviour, running intensity and MVPA for human beings [4, 10, 11]. With respects to neighbourhood perceptions, they are more often measured through the Likert scales [1, 12, 13]. Several research suggest that perceived neighbourhood safety is positively associated with healthy behaviours such as walking duration and leisure-time physical activity [12, 14, 15]; Good perceptions of neighbourhood aesthetics are conductive to leisure-time physical activity and MVPA [1, 9]. Despite a few efforts [16, 17], however, whether and how neighbourhood safety and aesthetics moderate the association between built environment and physical activity remains unclear in China.

Theoretically, built environment can affect physical activity differentially through the difference in perceptions of neighbourhood environment. It is documented that built environment has an effect on physical activity by environmental perception [12, 18]. Such perception is not only correlated to the objective environmental characteristics, but also in relation to one’s cognitive and affective factors, such as people’s preferences [19, 20]. Hence, there is usually a low agreement between the objective and perceived (subjective) measures of the built environment, documented both in social cognitive theory [21]and social ecological theory [22] and empirical findings [1, 23], which results in the difference in effects of built environment. On the other hand, people may have the same perception of environmental conditions, but such perceived environment has differential effects on physical activity among population subgroups [1]. This also leads to a differential association between built environment and physical activity found in many studies.

Several attempts have examined the moderating roles of neighbourhood safety and aesthetics. In general, the number of studies is quite small, and there is no consistent evidence that good perceptions of neighbourhood safety and aesthetics can enhance the positive effect of built environment on people’s healthy behaviours. Some studies support the argument [1, 13, 17]. In particular, the International Physical Activity and Environment Network (IPEN) Adult study with 6822 respondents from 10 countries, suggested that high levels of perceived neighbourhood safety and aesthetics, are related to stronger associations of moderate-to-vigorous physical activity (MVPA) with the proportion of retail and civic land, distance to the nearest transport stop and land use mix [1].

By contrast, several research reported opposite moderation patterns [24, 25]. As indicated in the two cross-sectional studies in Baltimore and Seattle-King County of USA, the interaction between walkability and safety from crime is negatively associated with total MVPA [25]. Again, a Greater London study with 3684 respondents who participated in the Understanding Society Survey Wave 6, reported that the effect of hard space on the subjective well-being was positive in neighbourhoods with high safety, but negative in low-safety neighbourhoods [24]. There are also some studies indicating that there is no moderation effect of neighbourhood environment [9, 16, 17]. In particular, no significant effect of the interaction between pleasurability and walk score, was found in an American study combining the automated audit approach and street view images [17].

Hence, it remains unclear whether good perceptions of neighbourhood safety and aesthetics can enhance or diminish the beneficial effect of favourable built environment on people’s physical activity, due to the limited studies and inconsistent findings. Moreover, most studies are concentrated in the developed counties [1, 26], while few studies have examined the moderation effects of neighbourhood perception in developing countries. Such studies are highly in China, where is with its great specialty and significance but not receive sufficient attention. To be specific, firstly, different from the open community in most western cities, the phenomenon of gated community is very popular in Chinese cities [27]. Secondly, there are great differences in residential density, community management and social relation between Chinese and western cities [18]. Thirdly, as stated in review studies, the pattern of moderation effect on the association of built environment with physical activity and health status may vary across geographical regions [26]. Therefore, it is of great significance to understand the pattern of moderation effects in the Chinese context. However, such studies are quite limited in China.

To fill the gaps above, with data collected from 760 respondents in Fuzhou, China, this work aims to investigate whether and how perceived neighbourhood safety and aesthetics moderate the association between built environment and time for recreational walking. There are two-fold contributions. On the one hand, this study contributes to the literature on potential moderators of built environment-physical activity associations from a perspective of perceived neighbourhood environment. On the other hand, we enrich the studies of built environment-associated physical activity by examining the moderation effect of neighbourhood environment in the Chinese context where is seldom examined and its urban form, neighbourhood management and social relation ware quite different from those of western countries.

## Materials and methods

### Research area

**Fig. 1:**
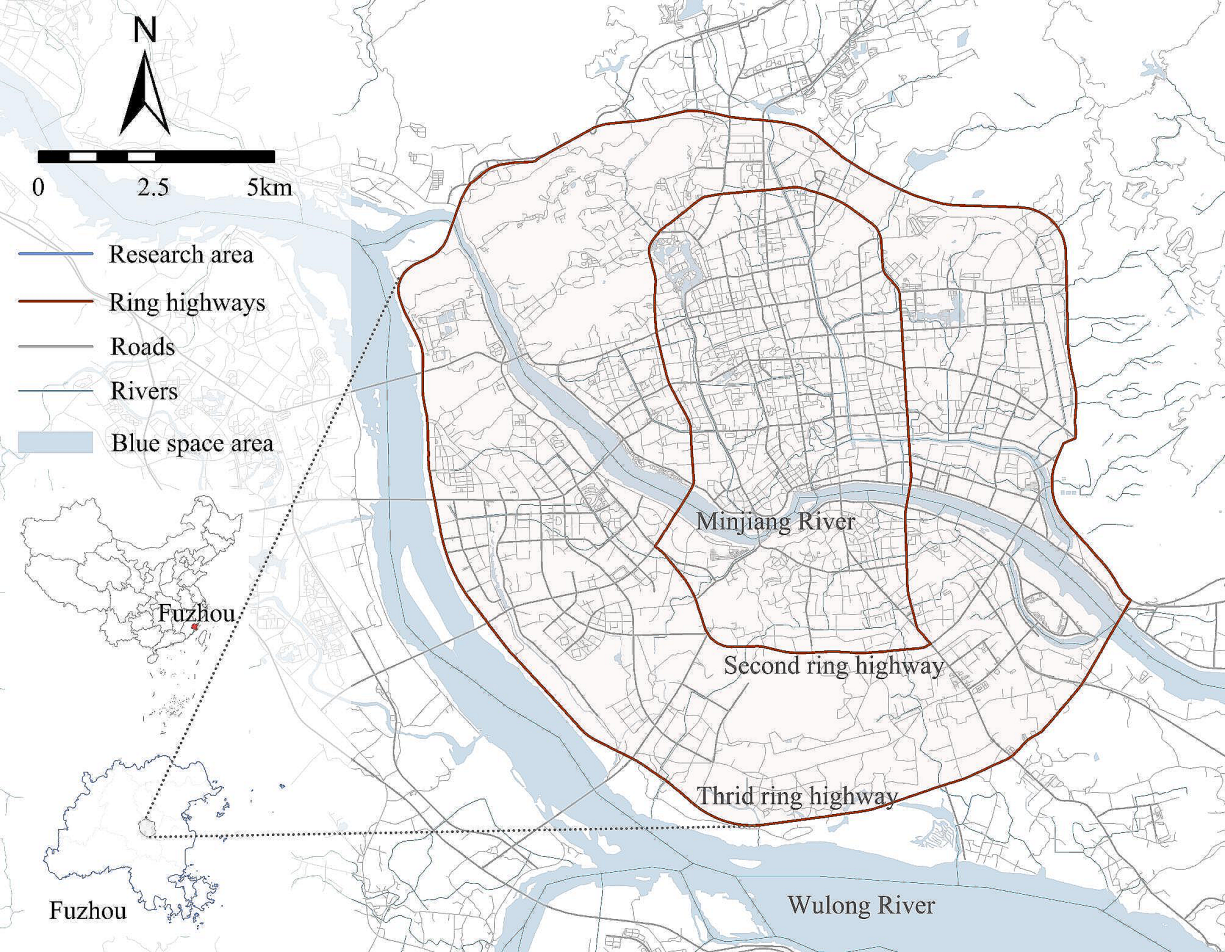
Spatial distribution of respondents across central areas of Fuzhou

We examined the moderation effects of neighbourhood safety and aesthetics in the built areas of Fuzhou City (Fig. 1). Fuzhou is the capital of Fujian Province, and one of the key second-tier cities in China, with the GDP of 1,292,847 billion in 2023. It covers a total area of about 11,090 km^2^ and a prefecture-level population of permanent residents nearly 842 million. Fuzhou is surrounded by mountains, divided by Minjiang River and Wujiang River, and characterized by favourable urban greenery. The moderate physical activity in Fuzhou is below that of 150 min per week recommended by the World Health Organization [26]. Perceived safety and aesthetics in average reached to 2.90 and 2.72 (4-point Likert Scale), respectively in this study. Moreover, research on Healthy City in China is mainly concentrated in its first-tier and main large cities [28, 29], while attention to the second-tier cities is not sufficient enough. Hence, Fuzhou is taken as the study case.

### Data

#### Physical activity

The variable of physical activity is time for recreational walking on a weekend day. It is defined as the total time of walking spent on recreational activities on a weekend day. According to the purpose, walking is usually divided into walking for recreation and transportation. We focus on the former one, because there is a difference in built environment correlates between the transport and recreational walking, as well as the debated findings of the built environment-recreational walking associations [4].

Data on time for recreational walking, were derived from the “Neighbourhood Environment and Residents’ Behaviour Survey”. It was conducted by the Lab of Urban Environment and Public Health at Fuzhou University from June to August, 2017. And the informed consent to participate has been obtained from all of the participants in the study. More details of questionnaire, data collection, processing about the survey can refer to [30]. Briefly, a pre-survey was performed online. Then, respondents were randomly selected within each street (township). The number of respondents were decided, according to the proportion of street’s (town’s) population reported in the Sixth National Population Census. Our questionnaires included the individual- and household-level socioeconomic information, perceived neighbourhood environment, personal preferences, healthy behaviours, as well as respondents’ physical and mental health. Finally, there were totally 2,000 respondents taking part in the survey, while 1712 questionnaires were collected. After removing questionnaires with missing and misclassified information, such as household address and perceived neighbourhood, totally 760 questionnaires (respondents) were used in this study.

#### Built environment elements

A network-based buffer with radii of 500 m, was created to measure built environment elements surrounding the respondents’ residential locations. It should be noted that there is the uncertain geographic context problem [31]. As in our previous work [30], we set the radii of the buffer area to 500 m, because some perceived built and social environment variables are measured within such a buffer area in the survey. Meanwhile, a buffer area with radii of 500 m is similar to that of the community–life cycles for 10 min in many Chinese cities [32]. Hence, a buffer with radii of 500 m was used to examine the moderation effects by neighbourhood safety and aesthetics in the present study. Data of the distribution of population, land use and road network, were provided by Fuzhou Planning and Design Institute, while points of interests (POIs) were derived from Baidu Maps (http://map.baidu.com).

**Fig. 2:**
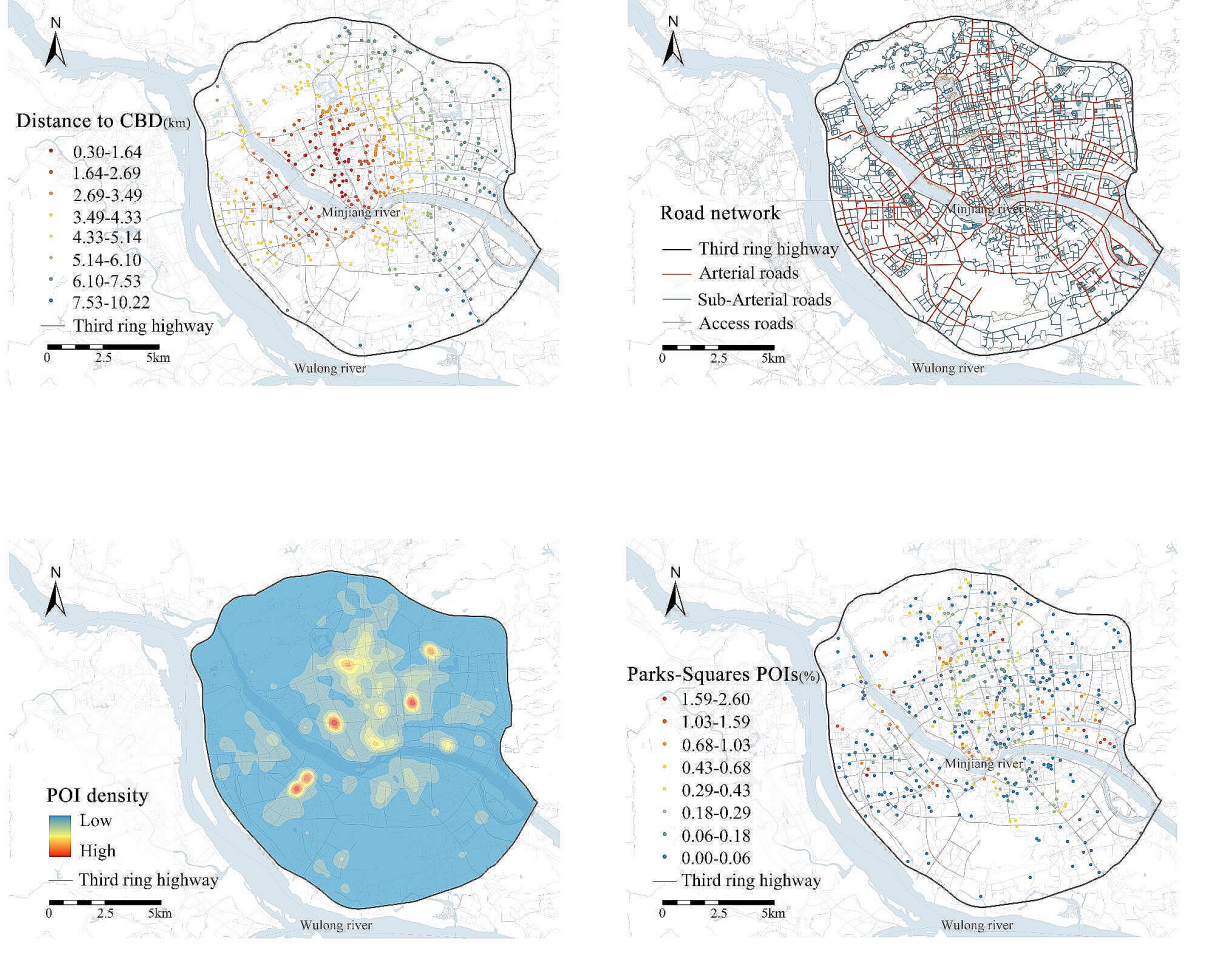
Spatial distributions of some built environment elements

Based on the 5D measurement of built environment and previous studies [8, 11], length of road network divided within a buffer, was used as the proxy of road density to measure road connectivity (i.e. road network design). Land use entropy index was employed to measure land use diversity, with a high value indicating a more mixed and diverse degree of land use types within a buffer. Land use entropy index was calculated in terms of the 20 types of POIs reflecting daily destinations [30].The geographic distribution of these variables is shown in Fig. 2.

#### Neighbourhood safety and aesthetics

Variables of neighbourhood safety and aesthetics were measured in a perceived way. Perception of safety, ranked as the second basic needs of human beings, not only promotes healthy behaviours [14, 33], but also contributes to human mental well-being and physical health [34, 35]. Neighbourhood safety and aesthetics can be measured both objectively and subjectively (i.e. perception). It is reported that perceived safety of a respondent is more likely to be associated with his/her physical activity [25, 36], especially for those who do not perceive threats from neighbourhoods but having high levels of crime rates. Similar to many studies [11, 13], therefore, neighbourhood safety and aesthetics were measured subjectively (i.e. perception) in this work.

**Fig. 3:**
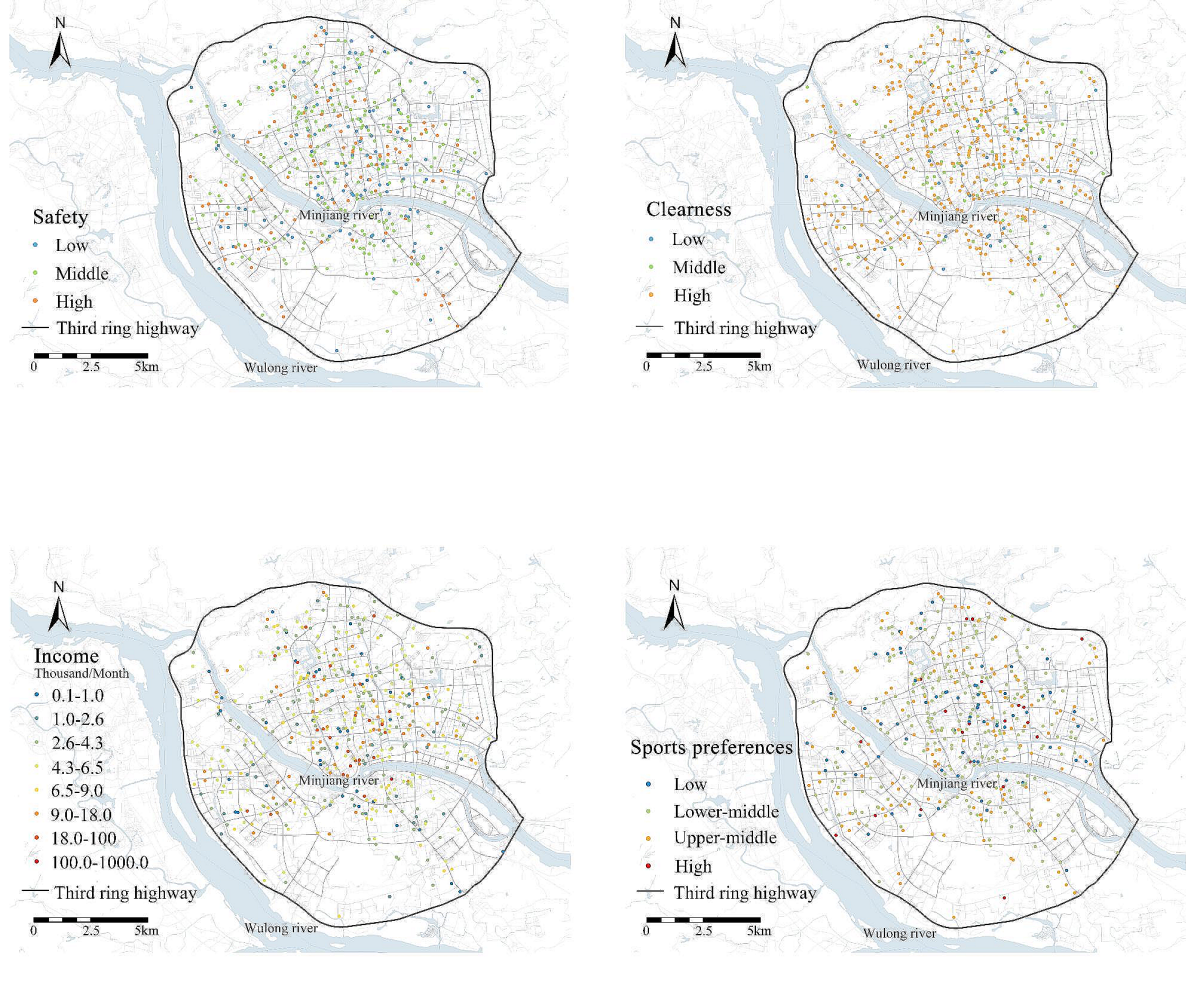
Spatial distributions of neighbourhood perception and socioeconomic factors

Data on perceived neighbourhood safety and aesthetics, were obtained from the “Neighbourhood Environment and Residents’ Behaviour Survey”. The two items were (1) Whether you worry about your personal or property injury (e.g. robbery and theft), when you walk in the area defined as within 500 m from home; (2) How you think the cleanness and sanitation of road pavements around your neighbourhood. The two items were measured using a 4-point Likert scale, with answers rating from “very worried” to “not worry at all”, and from “very clean” to “not clean at all”, respectively. To be consistent, answers for question concerning aesthetics were reversed. That is, a high score denotes a high level of perceived safety or aesthetics (Fig. 3).

#### Socioeconomic factors, location and other covariates

Four individual socioeconomic characteristics were included as cofounding variables. They are gender, age, the highest level of education acquired, and monthly income. Notably, the highest level of education is a dummy variable, with 1 denoting that a degree of undergraduate and above has been obtained. These variables are selected to control differences in time for recreational walking related to gender, age, economic status and education level according to previous studies [37, 38]. Regarding the impact of residential self-selection bias, similar to many studies [18, 32], individual preference to participating in sports was controlled in this work. The item was measured using a 4-point Likert scale, rating from do not like it at all (value = 1) to love it very much (value = 4). Notably, data on socioeconomic factors and personal preferences were derived from the “Neighbourhood Environment and Residents’ Behaviour Survey”. The variable of distance to CBD is operationalized as the distance between the respondent’s home and CBD (km).

### Statistical analysis

The Negative Binomial Regression Model (NBRM), was developed to examine moderation effects of neighbourhood safety and aesthetics. This model is suitable to examine the associations between the independent indicator and dependent variable, when the latter is in the format of count data. Compared to other models fitting count data such as Poisson regression model, the NBRM model is superior to its capacity of fitting the over-dispersed count data that are usually characterized by the variable’s variance greater than its mean. In our study, the outcome variable, i.e. time for recreational walking, is discrete in nature. Moreover, there is an overdispersion for the outcome variable, with its variance significantly greater than its mean. Hence, as in many prior studies [39–41], the NBRM model was selected in the present study.

Based on ecological models and previous studies [7, 32], we controlled for the impacts of location, socioeconomic factors, neighbourhood environment and personal preferences in the model. Among these neighbourhood environment factors included perceived aesthetics and safety. Personal preference contained individual preference to participating in sports. Referring to the criterion of previous studies [30, 42], there was no multi-collinearity among these variables, with all the values of variance inflation factor (VIF) lower than 5 in the present study.

To examine moderation effects, we added the two-way interaction terms between built environment elements and dummy variables of neighbourhood safety and aesthetics to the NBRM model, respectively. Considering the potential endogeneity for the moderating variables and interaction terms, we examined the effects of built environment on perceived safety and aesthetics using the Ordinal Logistic Regression model, respectively, adjusting for personal characteristics. According to the results (Table S1), there were no significant associations of perceived safety and aesthetics with each of the three moderators including road density, land use diversity, and accessibility to parks and squares.

Then, similar to studies exploring moderation effects [1, 43], a three-category division of the moderators was firstly employed to construct the interaction terms. To examine moderation effects in a robust way, a binary division was also used, instead of the solely division (three- or two-category) usually adopted in many previous studies [26, 44]. Regarding safety data with respondents’ response, 1 means you feel “worry very much” and 4 means you feel “not worry at all”. Similar to that of previous studies [45], from 1 to 2, 3 and 4, were divided as category 1 to 3 in the three-category division, respectively; from 1 to 2 and from 3 to 4, were divided as category 1 to 2 in the binary division. For aesthetics, the same division method was employed.

Notably, since there were high collinearities between dummy variable and its interaction with built environment element, safety (or aesthetics) dummy was not included in the NBRM model. Neighbourhood environmental moderators included the perceived neighbourhood safety and aesthetics. We targeted the three dimensions of built environment which may interact with the perceived neighbourhood environment, including road connectivity (i.e. road density), diversity (i.e. land use entropy index), as well as accessibility to parks and squares (i.e. proportion of park and square POIs).

Finally, two sensitivity analyses were conducted. Firstly, we tested whether the moderation effects of neighbourhood safety and aesthetics, are robust in the situation of further control of residential self-selection. Residential self-selection refers to a tendency that people choose the place of residence based on personal preferences, which may bias the association between built environment and physical activity [46]. For example, the difference in physical activities is likely to correlate with one’s personal preferences to sports instead of some built environment elements. Despite the use of personal preferences to sports as a way to control, it may still not be sufficient due to a large number of personality variables [7]. In China, Danwei or affordable housing are not self-selected by residents, but basically allocated by local governments. Hence, using samples of residents living in such types of housing accounting for 44.08% of the total samples, can well alleviate the bias in relation to residential self-selection, which thus produce more robust results of moderation roles. Secondly, the moderation effects were further tested using different measures of conceptually-comparable aspects of built environment. In this work, the number of road intersection within a buffer was used as the further proxy of road connectivity to examine the robustness of moderation effects.

## Results

### Descriptive analysis

Table 1 provides the summary statistics of respondent characteristics and neighbourhood environment elements. The mean and standard deviation of time for recreational walking on a weekend day, were 49.07 min and 74.92, respectively. This suggests a considerable variation in time spent on recreational walking among respondents. A substantial difference can also be found for perceived neighbourhood safety and aesthetics, with the standard deviations of 0.78 and 0.69, respectively. Regarding built environment elements, a similar pattern of results can be observed, especially for road connectivity (road length) and land use diversity (land use entropy index), with the standard deviations of 10.583 and 0.313, respectively. This demonstrates great variations in built environment characteristics across neighbourhoods.

**Table 1:** Descriptive statistics of respondents’ characteristics and neighbourhood environments

Variables	Mean	SD	Min	Median	Max
Recreational walking (minutes)	49.07	74.915	0	30	120
Perceived safety	2.900	0.784	1	3	4
Perceived aesthetics	2.788	0.691	1	3	4
Distance to CBD (km)	9.972	13.060	0.301	5.183	80.661
Road density ^a^	11.534	10.583	0.203	8.878	64.141
Road intersections	15.51	10.359	0	14	57
Sports POIs% ^b^	0.723	1.590	0	0.44	37.931
Parks-squares POIs ^b^	0.282	0.701	0	0	10
Land use diversity	2.205	0.313	0.693	2.289	2.672
Population density	1.381	1.238	0.005	0.990	4.698
POI density ^c^	12.095	10.032	1.005	9.859	86.238
Sports preferences	2.23	0.730	1	2	4
Sex (male = 1)	0.400	0.491	0	0	1
Age	31.32	9.268	18	31	72
Income (104 RMB) ^c^	0.744	3.658	0	0.5	5
Education	0.63	0.484	0	1	1

^a^ for value = original value/1000; ^b^ for value = original value×100; ^c^ for value = original value×10,000

### Moderation effects of perceived neighbourhood safety

The results of safety moderation role on the effects of road connectivity, accessibility to parks and squares and land use diversity, are presented in Tables 2, 3 and 4. Generally, neighbourhood safety positively moderated the association between road density (as proxy of road connectivity) and time for recreational walking. This implies that road density had stronger associations with time for recreational walking, when neighbourhood safety was perceived high than when neighbourhood safety was perceived low. Regarding the three-category division of neighbourhood safety, if there is a 0.01 km/km^2^ change in road density, then the shift in time for recreational walking was higher by 1.067 min (95%CI: -0.111, 2.245) and 2.361 min (95%CI: 0.501, 4.221) in the middle and high safety groups than in the low safety group. With regards to the binary division, the interaction between road density and safety dummy variable, was positively associated with time for recreational walking (β = 1.304, 95% CI: 0.162, 2.447).

**Table 2:** Safety moderation role: Road network design

	Three category		Binary division	
	β	95% CI	β	95% CI
Distance to CBD	0.015 ***	(0.006, 0.024)	0.014 ***	(0.005, 0.024)
Road density ^a^	1.249 **	(0.289, 2.210)	1.150 **	(0.208, 2.091)
Road intersections	-0.021	(-0.032, 0.010)	-0.020	(-0.031, -0.009)
Parks-squares POIs ^b^	0.035	(-0.144, 0.214)	0.030	(-0.149, 0.209)
Sports POIs%	-2.436	(-5.609, 0.737)	-2.449	(-5.646, 0.749)
Land use diversity	0.438 ***	(0.113, 0.763)	0.438 ***	(0.114, 0.763)
Population density	-0.099 **	(-0.194, -0.005)	-0.097 **	(-0.192, -0.002)
POI density ^b^	0.500	(-0.666, 1.667)	0.565	(-0.597, 1.727)
Sports preferences (2)	1.648 ***	(0.793, 2.504)	1.626 ***	(0.791, 2.462)
Sports preferences (3)	1.269 ***	(0.444, 2.093)	1.261 ***	(0.455, 2.067)
Sports preferences (4)	0.965 **	(0.123, 1.808)	0.954 **	(0.129, 1.779)
Sex	-0.138	(-0.330, 0.053)	-0.122	(-0.315, 0.071)
Age	0.005	(-0.005, 0.015)	0.005	(-0.005, 0.015)
Income	-0.045 ***	(-0.076, -0.014)	-0.044 ***	(-0.076, -0.012)
Education	0.396 ***	(0.190, 0.603)	0.406 ***	(0.198, 0.614)
Perceived aesthetics (2)	0.136	(-0.388, 0.661)	0.116	(-0.406, 0.639)
Perceived aesthetics (3)	-0.092	(-0.307, 0.123)	-0.102	(-0.317, 0.113)
Road density×Safety2 ^a^	1.067 **	(-0.111, 2.245)	1.304 **	(0.162, 2.447)
Road density×Safety3 ^a^	2.361 **	(0.501, 4.221)		
Log-likelihood ratio chi^2 b^	1.343 ***		1.314 ***	

* for *p* < 0.1, ** for *p* < 0.05 and *** for *p* < 0.01

^a^ for value = original value/100,000; ^b^ or value = original value×100

With regards to accessibility to parks and squares, the effect of proportion of parks and squares POIs was positively moderated by neighbourhood safety. In other words, a high level of neighbourhood safety magnifies the positive effect of accessibility to parks and squares. When the proportion of parks and squares POIs increased by 1%, the change in time for recreational walking was higher by 0.343 min (95%CI: 0.021, 0.664) and 0.355 min (95%CI: -0.067, 0.778) in the middle and high safety groups than in the low safety group, respectively. A similar pattern of results can be observed according to the binary division of neighbourhood safety. Specifically, there was a significant effect of the interaction between proportion of parks and squares POIs and safety dummy variable (β = 0.345, 95% CI: 0.034, 0.655).

**Table 3:** Safety moderation role: accessibility to parks and squares

	Three category		Binary division	
	β	95% CI	β	95% CI
Distance to CBD	0.014 ***	(0.005, 0.023)	0.014 ***	(0.005, 0.023)
Road density ^a^	1.773 ***	(0.669, 2.876)	1.769 ***	(0.670, 2.868)
Road intersections	-0.019	(-0.03, -0.008)	-0.019	(-0.03, -0.008)
Parks-squares POIs% ^b^	0.251**	(0.018, 0.520)	0.251**	(0.018, 0.520)
Sports POIs%	-2.349	(-5.694, 0.997)	-2.343	(-5.693, 1.007)
Land use diversity	0.440 ***	(0.121, 0.76)	0.440 ***	(0.121, 0.758)
Population density	-0.095 **	(-0.192, 0.001)	-0.095 **	(-0.192, 0.001)
POIs density ^b^	0.688	(-0.508, 1.884)	0.688	(-0.508, 1.884)
Sports preferences (2)	1.624 ***	(0.771, 2.476)	1.624 ***	(0.771, 2.476)
Sports preferences (3)	1.243 ***	(0.419, 2.068)	1.243 ***	(0.419, 2.068)
Sports preferences (4)	0.949 **	(0.106, 1.791)	0.948 **	(0.106, 1.79)
Sex	-0.118	(-0.312, 0.076)	-0.117	(-0.311, 0.077)
Age	0.005	(-0.005, 0.015)	0.005	(-0.005, 0.015)
Income	-0.043 **	(-0.077, -0.008)	-0.043 **	(-0.077, -0.008)
Education	0.399 ***	(0.192, 0.607)	0.399***	(0.192, 0.607)
Perceived aesthetics (2)	0.100	(-0.417, 0.616)	0.100	(-0.417, 0.616)
Perceived aesthetics (3)	-0.120	(-0.337, 0.096)	-0.120	(-0.337, 0.096)
Parks-squares POIs% ×Safety2 ^b^	0.343 **	(0.021, 0.664)	0.345 **	(0.034, 0.655)
Parks-squares POIs% ×Safety3 ^b^	0.355 *	(-0.067, 0.778)		
Log-likelihood ratio chi^2 b^	1.324 ***		1.325 ***	

* for *p* < 0.1, ** for *p* < 0.05 and *** for *p* < 0.01

^a^ for value = original value/100,000; ^b^ or value = original value×100

With respects to land use diversity, its effect was not significantly moderated by neighbourhood safety. In other words, the effect of land use entropy index (proxy of land use diversity) on the time for recreational walking, did not significantly vary among different levels of neighbourhood safety. In the situation of binary division, the interaction between entropy index and neighbourhood safety, was significantly correlated to time for recreational walking (β = 0.081 95%CI: -0.016, 0.179). However, as shown in Table 4, a similar pattern of results was not observed according to the three-category division, with only one interaction term having a significant effect.

**Table 4:** Safety moderation role: land use diversity

	Three category		Binary division	
	β	95% CI	β	95% CI
Distance to CBD	0.014 ***	(0.005, 0.023)	0.014 ***	(0.005, 0.023)
Road density ^a^	1.952 ***	(0.842, 3.063)	1.750 ***	(0.679, 2.821)
Road intersections	-0.020	(-0.031, -0.009)	-0.019	(-0.030, -0.008)
Parks-squares POI% ^b^	0.036	(-0.146, 0.217)	0.021	(-0.157, 0.199)
Sports POIs%	-2.119	(-5.310, 1.071)	-2.154	(-5.396, 1.088)
Land use diversity	0.383 **	(0.039, 0.728)	0.380 **	(0.035, 0.725)
Population density	-0.098 **	(-0.191, -0.004)	-0.092 **	(-0.186, 0.002)
POI density ^b^	0.549	(-0.595, 1.693)	0.594	(-0.542, 1.730)
Sports preferences (2)	1.683 ***	(0.828, 2.539)	1.634 ***	(0.799, 2.468)
Sports preferences (3)	1.295 ***	(0.471, 2.119)	1.255 ***	(0.450, 2.059)
Sports preferences (4)	1.015 **	(0.173, 1.856)	0.957 **	(0.136, 1.779)
Sex	-0.136	(-0.328, 0.056)	-0.121	(-0.313, 0.071)
Age	0.004	(-0.006, 0.014)	0.004	(-0.006, 0.014)
Income	-0.045 ***	(-0.076, -0.014)	-0.044 ***	(-0.076, -0.011)
Education	0.357 ***	(0.149, 0.564)	0.398 ***	(0.190, 0.606)
Perceived aesthetics (2)	0.170	(-0.367, 0.706)	0.130	(-0.403, 0.663)
Perceived aesthetics (3)	-0.088	(-0.302, 0.126)	-0.104	(-0.318, 0.111)
Land use diversity×Safe2	0.043	(-0.061, 0.147)	0.081 *	(-0.016, 0.179)
Land use diversity×Safe3	0.184 ***	(0.060, 0.308)		
Log-likelihood ratio chi^2 b^	1.397 ***		1.294 ***	

* for *p* < 0.1, ** for *p* < 0.05 and *** for *p* < 0.01

^a^ for value = original value/100,000; ^b^ for value = original value × 100

### Moderation effects of perceived neighbourhood aesthetics

Tables 5, 6 and 7 exhibits the results of aesthetics moderation role on the effect of accessibility to parks and squares, road connectivity and land use diversity. Generally, neighbourhood aesthetics positively moderated the effect of the proportion of parks and squares POIs. It means that recreational walking would be better promoted, when neighbourhoods were with better accessibility to parks and squares, and also with high aesthetics perception. When there was a 1% increase in proportion of parks and squares POIs, the change in time for recreational walking was higher by 0.500 min (95%CI: -0.135, 1.055) and 0.567 min (95%CI: -0.029, 1.046) in the middle and high aesthetics groups than in the low aesthetics group, respectively. A similar pattern of results can be observed in the situation of aesthetics’ binary division. Specifically, the interaction between proportion of parks and squares POIs and aesthetics dummy variable was positive, with the coefficient by 0.556 (95% CI: -0.038, 1.033).

**Table 5:** Aesthetics moderation role: accessibility to parks and squares

	Three category		Binary division	
	β	95% CI	β	95% CI
Distance to CBD	0.013 ***	(0.007, 0.020)	0.013 ***	(0.007, 0.020)
Road density	1.890 ***	(0.779, 3.058)	1.894 ***	(0.785, 3.061)
Road intersections	-0.019	(-0.029, -0.008)	-0.019	(-0.029, -0.008)
Parks-squares POIs%	0.514 ***	(0.072, 0.975)	0.514 ***	(0.073, 0.975)
Sports POIs%	-2.692	(-6.666, 3.126)	-2.629	(-6.624, 3.217)
Land use diversity	0.409 **	(0.131, 0.670)	0.409 **	(0.130, 0.669)
Population density	-0.108 **	(-0.189, -0.026)	-0.109 **	(-0.189, -0.027)
POIs density	0.622	(-0.324, 1.609)	0.616	(-0.329, 1.603)
Sports preferences (2)	1.641 ***	(1.163, 2.089)	1.646 ***	(1.168, 2.093)
Sports preferences (3)	1.235 ***	(0.789, 1.644)	1.239 ***	(0.793, 1.647)
Sports preferences (4)	0.959 **	(0.509, 1.373)	0.962 ***	(0.513, 1.376)
Sex	-0.130	(-0.292, 0.032)	-0.127	(-0.289, 0.034)
Age	0.004	(-0.005, 0.013)	0.004	(-0.005, 0.013)
Income	-0.045 ***	(-0.101, -0.002)	-0.045***	(-0.101, -0.002)
Education	0.349 ***	(0.186, 0.514)	0.349 ***	(0.186, 0.514)
Perceived safety (2)	-0.356 **	(-0.586, -0.128)	-0.358 **	(-0.588, -0.13)
Perceived safety (3)	-0.319 **	(-0.517, -0.126)	-0.320 **	(-0.517, -0.127)
Parks POIs% × Aesthetics2 ^b^	0.500 **	(-0.135, 1.055)	0.556 ***	(-0.038, 1.033)
Parks-squares POIs% × Aesthetics 3 ^b^	0.567 ***	(-0.029, 1.046)		
Log-likelihood ratio chi^2 b^	1.399 ***		1.397 ***	

* for *p* < 0.1, ** for *p* < 0.05 and *** for *p* < 0.01

^a^ for value = original value / 1000; ^b^ for value = original value × 1000

Regarding road connectivity, neighbourhood aesthetics did not moderate the association of time for recreational walking with road density. There were no significant effects of the interactions between road density and aesthetics dummy variables, according to the three-category or binary divisions of neighbourhood aesthetics. With respects to land use diversity, no significant moderation effect of neighbourhood safety was observed. Specifically, the association between entropy index and time for recreational walking did not significantly vary across differential aesthetics levels in the situation of three-category division. A similar pattern of results can be found according to the binary division, with an insignificant effect of the interaction between entropy index and aesthetics dummy variable.

**Table 6:** Aesthetics moderation role: Road network design

	Three category		Binary division	
	β	95% CI	β	95% CI
Distance to CBD	0.013 ***	(0.007, 0.020)	0.013 ***	(0.007, 0.020)
Road density	0.171	(-2.743, 3.319)	-0.131	(-3.009, 2.986)
Density of road intersections	-0.019	(-0.029, -0.008)	-0.019	(-0.029, -0.008)
Parks-squares POIs%	0.027	(-0.087, 0.153)	0.024	(-0.090, 0.150)
Sports POIs%	-2.632	(-6.633, 3.224)	-2.635	(-6.629, 3.210)
Land use diversity	0.426 **	(0.149, 0.685)	0.427 **	(0.151, 0.686)
Population density	-0.111 **	(-0.191, -0.029)	-0.108	(-0.188, -0.026)
POIs density	0.534	(-0.419, 1.531)	0.639	(-0.307, 1.628)
Sports preferences (2)	1.645 ***	(1.138, 2.122)	1.586 ***	(1.086, 2.055)
Sports preferences (3)	1.250 ***	(0.777, 1.688)	1.191 **	(0.725, 1.619)
Sports preferences (4)	0.979 **	(0.505, 1.419)	0.918 **	(0.452, 1.348)
Sex	-0.125	(-0.288, 0.038)	-0.123	(-0.286, 0.040)
Age	0.004	(-0.005, 0.013)	0.004	(-0.005, 0.013)
Income	-0.044 ***	(-0.099, -0.001)	-0.045 ***	(-0.100, -0.002)
Education	0.340 ***	(0.176, 0.505)	0.347 ***	(0.183, 0.511)
Perceived safety (2)	-0.363 **	(-0.595, -0.132)	-0.345 **	(-0.575, -0.116)
Perceived safety (3)	-0.330 ***	(-0.530, -0.135)	-0.310 **	(-0.507, -0.117)
Road density × Aesthetics2 ^b^	2.653	(-0.400, 5.491)	2.036	(-0.880, 4.688)
Road density× Aesthetics 3 ^b^	1.681	(-1.281, 4.387)		
Log-likelihood ratio chi^2 b^	1.400 ***		1.382 ***	

* for *p* < 0.1, ** for *p* < 0.05 and *** for *p* < 0.01

^a^ for value = original value / 1000; ^b^ for value = original value × 1000

**Table 7:** Safety moderation role: land use diversity

	Three category		Binary division	
	β	95% CI	β	95% CI
Distance to CBD	0.013 ***	(0.007, 0.020)	0.013 ***	(0.007, 0.020)
Road density ^a^	1.952 ***	(0.839, 3.122)	1.894 ***	(0.786, 3.060)
Road intersections	-0.018	(-0.029, -0.008)	-0.019	(-0.029, -0.008)
Parks-squares POIs%	0.031	(-0.084, 0.157)	0.024	(-0.091, 0.149)
Sports POIs%	-2.870	(-6.889, 2.991)	-2.671	(-6.642, 3.126)
Land use diversity	0.405 **	(0.099, 0.704)	0.408 **	(0.102, 0.705)
Population density	-0.111 **	(-0.191, -0.029)	-0.111 **	(-0.192, -0.030)
POIs density	0.523	(-0.427, 1.514)	0.620	(-0.324, 1.605)
Sports preferences (2)	1.751 ***	(1.263, 2.212)	1.675 ***	(1.197, 2.125)
Sports preferences (3)	1.343 ***	(0.889, 1.762)	1.275 ***	(0.829, 1.685)
Sports preferences (4)	1.068 ***	(0.610, 1.492)	0.995 **	(0.546, 1.408)
Sex	-0.137	(-0.300, 0.025)	-0.138	(-0.301, 0.024)
Age	0.004	(-0.005, 0.013)	0.004	(-0.005, 0.013)
Income	-0.044 ***	(-0.101, -0.002)	-0.044 ***	(-0.100, -0.002)
Education	0.334 ***	(0.171, 0.499)	0.344 ***	(0.181, 0.509)
Perceived safety (2)	-0.374 ***	(-0.607, -0.141)	-0.357 **	(-0.589, -0.126)
Perceived safety (3)	-0.335 ***	(-0.534, -0.140)	-0.313 **	(-0.510, -0.120)
Land use diversity ×Aesthetics2	0.066	(-0.106, 0.228)	0.021	(-0.140, 0.170)
Land use diversity ×Aesthetics3	0.000	(-0.163, 0.152)		
Log-likelihood ratio chi^2 b^	1.386 ***		1.363 ***	

* for *p* < 0.1, ** for *p* < 0.05 and *** for *p* < 0.01. ^a^ for value = original value / 1000;

### Sensitivity analysis

#### Further control of residential self-selection bias

**Fig. 4:**
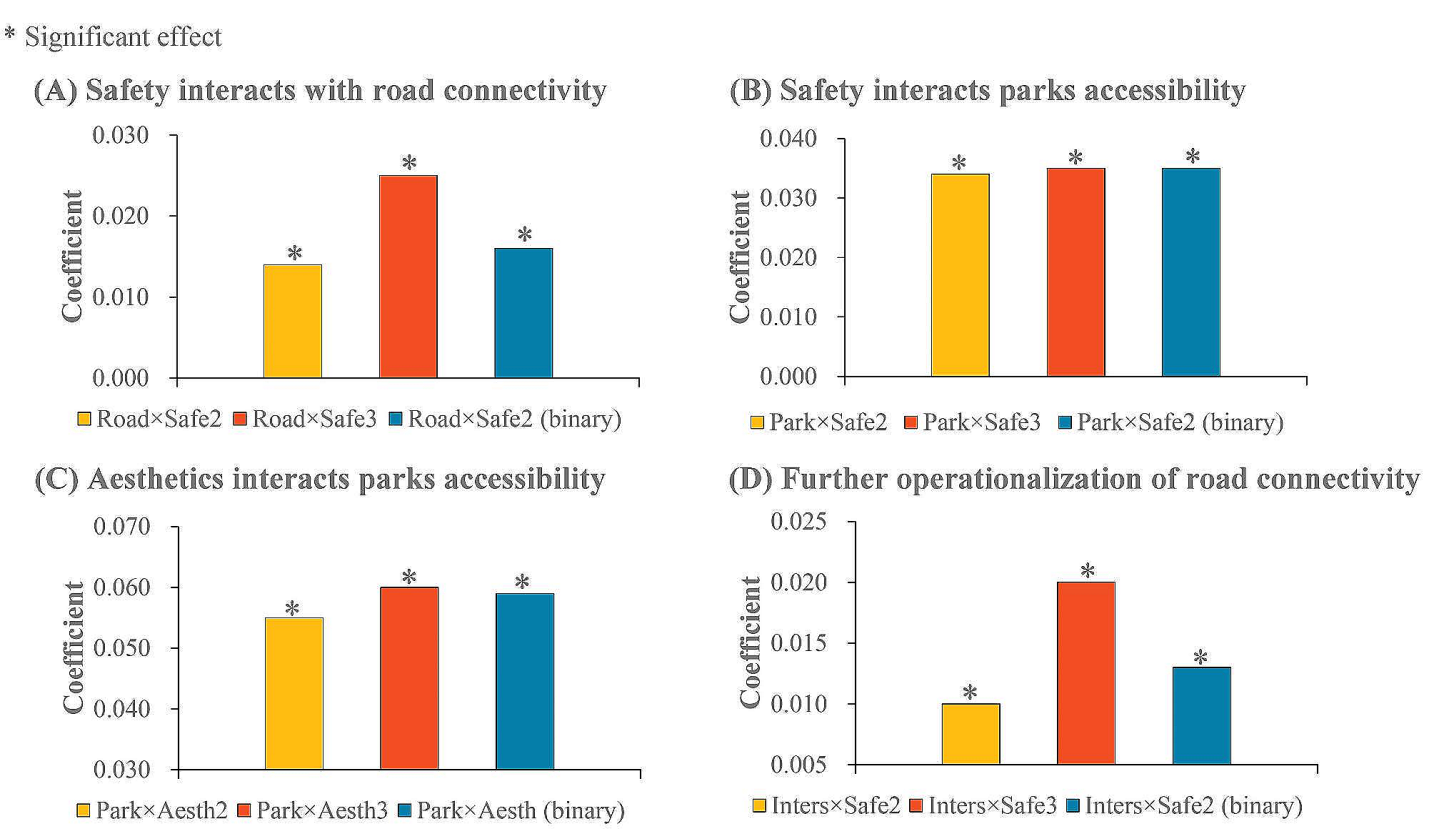
Sensitivity analysis of safety and aesthetics moderation effects

Figure 4(A), Fig. 4(B) and Fig. 4(C) present the results of sensitivity analysis of moderation effects to the further control of residential self-selection. Generally, safety moderating roles on the effects of road density and proportion of parks and squares POIs, were robust using samples living in Danwei or affordable housing neighbourhoods to control residential self-selection. As shown in Fig. 4(A), when road density increased by 0.01 km/km2, then the elevation in time for recreational walking was higher by 1.395 min (95%CI: 0.258, 2.533) and 2.511 min (95%CI: 0.657, 4.365) in the middle and high safety groups than in the low safety group, according to the three-category division; The interaction between road density and safety dummy variable, was also positive and significant in the situation of binary division (β = 1.575, 95%CI: 0.460, 2.690).

A similar pattern of results can be observed for the proportion of parks and squares POIs according to the three-category and binary divisions of neighbourhood safety (Fig. 4(B)). With regards to aesthetics (Fig. 4(C)), its moderation role on the effect of proportion of parks and squares POIs was still significant and positive. That is, when samples were limited to those living in Danwei or affordable housing, there were positive effects of the interaction(s) between aesthetics dummy variable(s) and proportion of parks and squares POIs. In particular, if the proportion of parks and squares POIs increased by 1%, the change in time for recreational walking was higher by 0.346 min (95%CI: 0.041, 0.651) in the high aesthetics group than that of the low aesthetics group.

#### Density of road intersections as a proxy of road connectivity

The results of sensitivity analysis using road intersection density as a proxy of road connectivity, are shown in Fig. 4(D). In general, the moderation effect of neighoborhood safety was still positive and significant. The interaction between road intersection density and safety dummy variable, was positively associated with time for recreational walking, with the coefficient by 0.013 (95%CI: 0.002, 0.024) in the situation of binary division (Fig. 4(D)). A similar pattern of results can be observed according to the three-category division. That is, there were positive effects of the two interaction terms, with coefficients by 0.010 (95%CI: -0.001, 0.021) and 0.020 (95%CI: 0.006, 0.033), respectively (Fig. 4(D)).

## Discussions

Understanding moderation roles of neighbourhood characteristics, is of great significance to make efficient interventions in built environment to better promote physical activity. However, it still remains unclear whether and to which extent good neighbourhood perception (especially for safety and aesthetics) can enhance the benefit of favourable built environment. In addition, studies exploring neighbourhood moderating roles, are less understood in developing countries, especially in Chinse cities.

To the best of our knowledge, this is one of the few studies examining neighbourhood safety and aesthetics moderation effects on built environment-physical activity associations in the Chinese context. On the one hand, this work contributes to the literature on potential moderators of built environment-physical activity associations from a perspective of neighbourhood environment. On the other hand, findings are derived from a developing setting where its urban form, neighbourhood management and social relation are quite different from those of western countries [18, 47], thus enrich the findings and promote further development of Ecological Models of Health Behavior [7].

We found a positive moderation role of neighbourhood safety on the effects of road connectivity and access to parks and squares. This implicates that to better promote residents’ physical activities, neighbourhoods with dense and well-interconnected road network and high accessibility to recreational facilities (parks and squares), need to be safe enough at the same time. It is still not clear how neighbourhood safety magnifies the benefits of road connectivity and destination accessibility. Neighbourhoods with dense road network, are usually with high availability and accessibility to walking and recreational facilities, especially for the blue and green space [1, 3, 17]. A high level of perceived neighbourhood safety, can encourage and promote the more and frequent walking activities surrounding or within these facilities [17, 25].

In our study, physical activity is measured during summertime when temperature is usually very high in many Chinese cities. Particularly, Fuzhou, famous for its extremely high temperature in summer, has become one of the hottest cities in China since the 21st century, and thus been named for “Furnace City” [48]. People may be more likely to conduct more and frequent recreational activities surrounding the neighbourhood at night than in the daytime on the weekend [10], especially for activities conducted in parks and squares partly due to a comfortable temperature during the night. It is documented that safety is a key determinant of people’s activities [14, 33], especially at night. Hence, if people feel the neighbourhood safe, they may spend more time and conduct more and frequent activities at such places, especially for recreational walking activities with non-purposes.

This is expected and consistent with findings from many studies. A Hong Kong study demonstrates that safe neighbourhoods with high accessibility to recreational and public facilities, promote the elders’ time spent on recreational walking in urban Hong Kong [9, 16]. Similarly, a world-wide study across 10 counties, showed that associations of MVPA (i.e. moderate to vigorous physical activities) with the ratio of retail/civic land and/or accessibility to transport stop, are stronger in neighbourhoods with a high level of perceived safety or aesthetics [1]. Also, a study conducted in two American counties, suggested that the interaction between walkability and neighbourhood safety is positively correlated with residents’ total MVPA [25]. Despite a positive moderation effect found in these studies, further research is highly in need to determine how neighoborhood environment moderates the effects of built environment elements.

We found a greater moderation role by aesthetics than safety on the effect of accessibility to parks and squares, which is seldom reported. Most previous studies focus on the effect of built environment elements on physical activity [42], and few efforts have examined safety and aesthetics as key moderators [24, 25]. As an extension, this work is one of the few studies to explore neighbourhood safety and aesthetics as potential moderators and compare their moderation effects. Specifically, the absolute values of changes in two interaction terms were 0.500 (95%CI: -0.135, 1.055) and 0.567 (95%CI: -0.029, 1.046) for neighbourhood aesthetics, which are higher than those of the two interaction terms at 0.343 (95% CI: 0.021, 0.664) and 0.355 (95% CI: -0.067, 0.778) for neighbourhood safety, respectively. A similar pattern of results can be observed according to each of the binary division of safety and aesthetics moderators, with a larger moderation effect by neighbourhood aesthetics (Tables 2 and 5).

The findings derived from the present study have several implications for urban planning and design. Firstly, we found that there are positive associations of time for recreational walking with road density and land use diversity. This underscores that to encourage physical activity for recreational purpose, urban planning can pay more attention to improve road network density and the mixture of land use in plans such as Master Plan. Secondly, we found positive moderation effects of neighbourhood safety on the associations of time for recreational walking with road connectivity and accessibility to parks and squares. This further highlights that to better promote recreational physical activities, interventions in built environment should not only pay to the optimization for neighbourhood road network, but also target the improvement of perceived neighbourhood safety, such as improving the greenery and open space.

Several limitations and future directions should be noted in this work. Firstly, as in many studies [3, 25, 42], the use of a buffer area with the radii by 500 m as neighbourhoods (analysis unit), suffers from the uncertain geographic context problem [31]. In our work, many built and social environmental elements are measured within such an area (radii by 500 m). Moreover, the buffer area created is also similar to that of the Chinese community–life cycles for 10 min [32]. However, sufficient attention should still be paid, and if data on neighbourhoods defined with different radiis are available, it would be more preferable.

Secondly, the outcome variable of time for recreational walking is measured on a weekend day, our findings may not be suitable on weekdays, due to differential patterns of built environment and physical activity correlations between weekday and weekend days as reported [10]. However, the availability of physical activity on weekdays potentially allow for the separation of physical activity from recreational and transportation purposes, which can widely open up the database to allow for studies evaluating built environment impacts on physical activity for transportation purpose. If physical activity data on both weekday and weekend are available, it would be much more preferable. Thirdly, future research can broaden the scope of potential moderators, especially for socioeconomic and psychosocial characteristics [35, 38], because of the limited and inconclusive evidence derived from existing studies.

Thirdly, this is a cross-sectional study in nature. Hence, as in many built environment-associated health studies [1, 11, 25], it is hard to infer the causal associations. The findings on the moderation role of neighbourhood safety and aesthetics in the present study may be sensitive, when a longitudinal study design is used. Thus, sufficient concerns should be raised to such a limitation and longitudinal data on physical activity are recommended for further examination. Fourthly, there still may be selection bias due to the removal of some questionnaires with missing and misclassified information, although a sample of 760 residents is a reasonable sample size and representative of the broader population of Fuzhou City, China. Hence, such a potential limitation still should receive sufficient attention.

Fifthly, as in many built environment-associated physical activity studies [26, 42, 49], since the variable of time for recreational walking was measured through a self-reported way, which is susceptible to recall and social desirability bias, thus leading to the potential for errors introduced by such a measurement in the present study. Finally, future studies can measure neighbourhood aesthetics and safety objectively through the Microscale Audit of Pedestrian Streetscapes (MAPS) tool [50–52], and then compare the results from the moderation effects of the two variables measured objectively and subjectively. Objective measurements of safety and aesthetics, in combination with perceived ones, would contribute to an in-depth and systematic understanding of the moderation effects of these two factors.

## Conclusions

Compared to those with low perceived neighoborhood safety, associations of time for recreational walking with road connectivity and accessibility to parks and squares are stronger for residents with high perceived neighbourhood safety. There is a positive moderation role by perceived neighbourhood aesthetics on the effect of accessibility to parks and squares. To our knowledge, this is one of the few studies exploring safety and aesthetics moderation role on built environment-physical activity associations in the Chinese context. The findings highlight that to well promote physical activity, neighbourhoods with a dense and highly-connected road network and high destination accessibility, should also be safe and aesthetic enough. Future studies should consider safety- and/or aesthetic-specific differences in estimates of built environment effects.

### Electronic supplementary material

Below is the link to the electronic supplementary material.

Supplementary Material 1

## Data Availability

The datasets during and/or analysed during the current study available from the corresponding author on reasonable request.
