# Supplementary material for: Can good neighbourhood perception magnify the positive effect of favourable built environment on recreational walking in China?
Source: BMC Public Health. 2024 Jul 29;24:2035. doi: 10.1186/s12889-024-19539-x (PMC11287862; doi:10.1186/s12889-024-19539-x)
Supplement: Supplementary file 1 — Supplementary Material 1 [file 12889_2024_19539_MOESM1_ESM.docx]

Table S1. Effects of built environment on perceived safety and aesthetics

|  | Perceived safety | | Perceived aesthetics | |
| --- | --- | --- | --- | --- |
|  | β | 95% CI | β | 95% CI |
| Distance to CBD ^b^ | -0.032 | (-1.224, 1.160) | -0.002 | (-0.014, 0.011) |
| Road density ^a^ | -0.015 | (-0.077, 0.046) | 0.050 | (-0.015, 0.115) |
| Road intersections | -0.004 | (-0.020, 0.013) | 0.006 | (-0.011, 0.023) |
| Parks-squares POIs ^b^ | -3.360 | (-22.732, 16.011) | -2.777 | (-22.725, 17.171) |
| Sports POIs% | -3.915 | (-12.332, 4.502) | -3.075 | (-11.607, 5.457) |
| Land use diversity | 0.253 | (-0.227, 0.733) | 0.136 | (-0.355, 0.627) |
| Population density | -0.184** | (-0.334, -0.035) | -0.082 | (-0.238, 0.073) |
| POI density ^b^ | 0.339*** | (0.158, 0.519) | 0.065 | (-0.121, 0.249) |
| Sex | 0.004 | (-0.282, 0.290) | -0.158 | (-0.460, 0.144) |
| Age | 0.020** | (0.005, 0.035) | -0.005 | (-0.021, 0.011) |
| Income | -0.033 | (-0.078, 0.013) | 0.425*** | (0.154, 0.696) |
| Education | 0.126 | (-0.168, 0.419) | -0.091 | (-0.397, 0.214) |
| Log-likelihood ratio chi^2 b^ | 1.699*** |  | 1.557** |  |

* for *p* <0.1, ** for *p* <0.05 and *** for *p* <0.01.

^a^ for value = original value / 1000; ^b^ for value = original value × 1000.
